# Femoral neck fractures in patients with prior lower-limb amputation: a two-case series and narrative review of surgical decision-making

**DOI:** 10.3389/fsurg.2026.1949434

**Published:** 2026-10-07

**Authors:** Dan Li, Haiyang Yu, Wanrong Yang

**Affiliations:** 1 Department of Orthopedics, Fuyang People’s Hospital Affiliated to Anhui Medical University, Fuyang, Anhui, China; 2 Department of Orthopedics, Clinical Research Center for Spinal Deformity of Anhui Province, Fuyang, Anhui, China

**Keywords:** cannulated compression screw, femoral neck fracture, individualized surgical decision-making, lower-limb amputation, total hip arthroplasty

## Abstract

**Background:**

Femoral neck fractures in patients with pre-existing lower-limb amputation are rare clinical conditions that pose unique technical challenges and surgical decision-making dilemmas for orthopedic surgeons. Altered hip biomechanics, reduced bone mineral density, and dependence on prosthetic ambulation complicate preoperative planning and postoperative rehabilitation, and no unified therapeutic consensus has been established in clinical practice ( 1– 5).

**Case presentation:**

We report two distinct clinical cases of femoral neck fractures in amputees managed with different surgical strategies. The first patient was a 55-year-old male with ipsilateral transfemoral amputation who sustained a minimally-displaced Garden II femoral neck fracture and underwent closed reduction combined with cannulated compression screw (CCS) internal fixation. The second patient was a 70-year-old male who experienced left transtibial amputation in a mining accident in 1984; he sustained a fully-displaced Garden IV femoral neck fracture and received primary total hip arthroplasty (THA).

Total follow-up was 12 months for Case 1 and 6 months for Case 2. Both patients achieved satisfactory short-term functional recovery, with validated functional scores recorded at final follow-up, and no major perioperative complications occurred.

**Conclusions:**

This two-case series combined with a narrative review highlights the need for individualized surgical strategies for amputees with femoral neck fractures. Based on our limited clinical observations, internal fixation may be suitable for younger patients with minimally-displaced fractures and preserved pre-injury hip function, whereas THA may yield favorable functional outcomes in elderly patients with completely-displaced fractures. The proposed treatment algorithm should be regarded only as experience-based guidance rather than universal clinical recommendations.

## Introduction

Femoral neck fractures are common geriatric injuries associated with substantial morbidity and long-term mortality (6). However, femoral neck fractures occurring after lower-limb amputation remain uncommon and present distinct surgical challenges for orthopedic practitioners (7). These patients demonstrate abnormal hip load distribution, generalized osteopenia, demanding intraoperative positioning requirements, and an urgent need for early mobilization to resume prosthetic ambulation. All of these factors complicate surgical planning and postoperative care.

Prior publications have reported acceptable outcomes following either internal fixation or arthroplasty in amputee patients, yet no standardized therapeutic algorithm exists. Most relevant literature consists of isolated single-case reports lacking comparative surgical experience (5, 8, 9).

### Narrative review methods

We performed a narrative literature review by searching PubMed, Embase, and CNKI using the search terms: “femoral neck fracture”, “transfemoral amputation”, “transtibial amputation”, “above-knee amputation”, “below-knee amputation”, “hip arthroplasty”, “cannulated screw fixation”. The search covered publications from January 2010 to June 2026. Eligible sources included case reports and case series focusing on femoral neck fractures in patients with pre-existing lower-limb amputation. Reviews, animal studies, and editorials were excluded. Relevant clinical variables from eligible studies were synthesized and discussed in the subsequent text.

Herein, we describe two amputees with femoral neck fractures treated with CCS fixation and THA, respectively. We integrate our intraoperative techniques and follow-up results with published evidence to propose a stratified, individualized decision-making framework for these rare injuries. This framework reflects single-center clinical experience and is not intended as formal clinical guidelines.

## Case presentation

### Case 1: cannulated compression screw fixation for Garden II femoral neck fracture

A 55-year-old male patient with a history of ipsilateral transfemoral amputation (time since amputation: 9 years; stump length: 25 cm; stump condition: intact soft-tissue coverage without skin ulceration; comorbidities: coronary heart disease, type 2 diabetes mellitus; ASA physical status: II; smoking-medication history: no smoking history and no regular long-term medication) presented with hip pain following a fall. Pelvic radiographs, axial hip CT, and stump radiographs confirmed a minimally-displaced Garden II femoral neck fracture, along with imaging evidence of prior transfemoral amputation (Figure 1).

**Figure 1:**
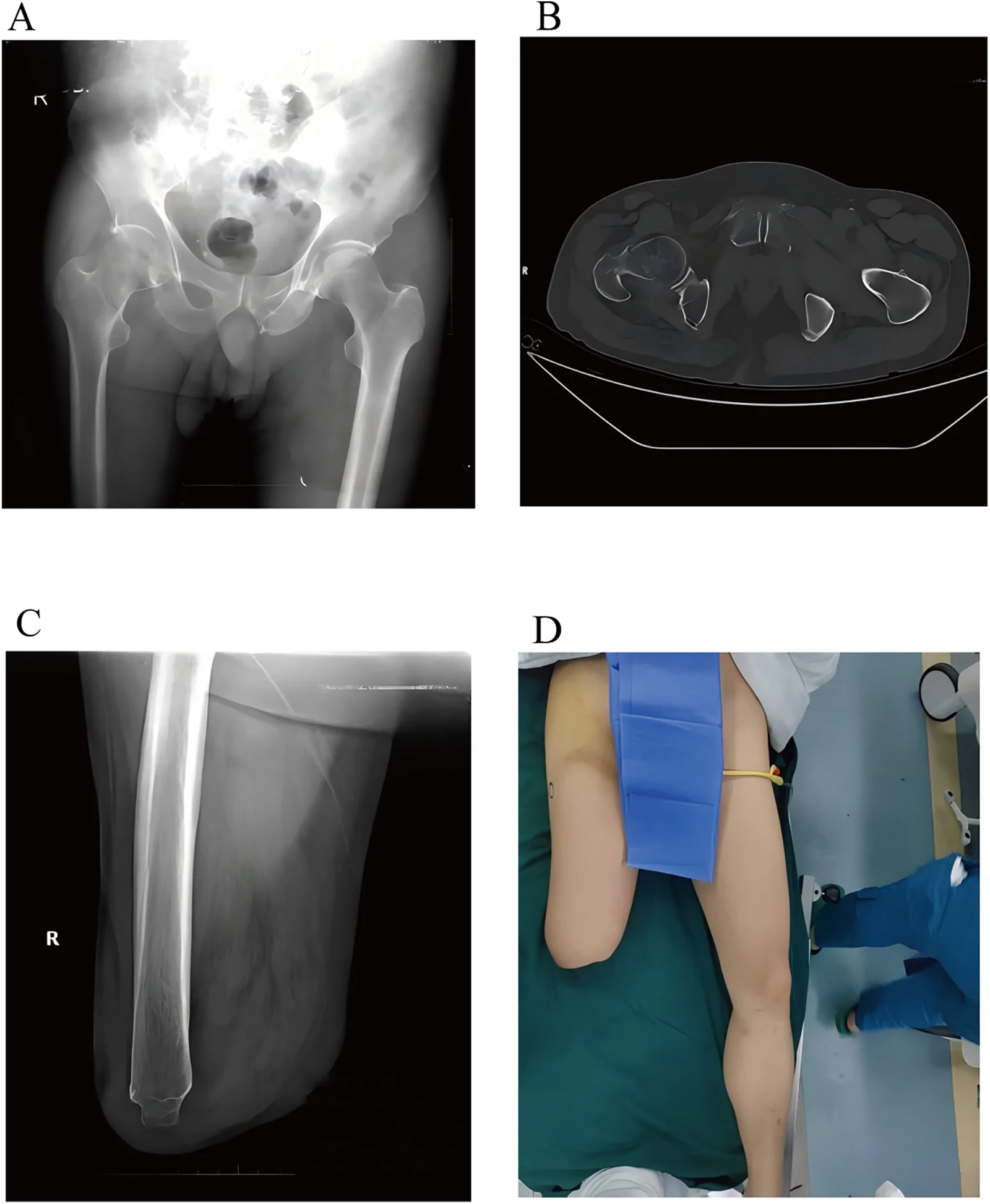
Preoperative imaging examinations of case 1. **(A)** Anteroposterior pelvic radiograph demonstrating minimally-displaced Garden II right femoral neck fracture; **(B)** Axial computed tomography scan of the hip confirming fracture morphology and displacement magnitude; **(C)** Radiograph of the ipsilateral transfemoral amputation stump; **(D)** Clinical photograph showing the appearance of the residual lower limb before surgery.

The patient maintained satisfactory pre-injury hip function and high functional demands. Bone quality was characterized as normal bone mineral density. The interval from injury to surgery was 4 days.

Closed reduction was performed on a fracture traction table using our self-designed customized traction device, followed by percutaneous cannulated compression screw internal fixation. The patient was positioned supine; the contralateral intact lower limb was secured in the lithotomy position, and a G-arm fluoroscope was positioned on the operative side. The residual stump was wrapped with sterile drapes and traction bandages to achieve stable traction for fracture reduction (Figure 2). Under real-time fluoroscopic guidance, three 6.5-mm cannulated compression screws (implant specifications: screw lengths: 85 mm, 80 mm, and 95 mm) were inserted in an inverted-triangular configuration to stabilize the femoral neck fracture (Figure 3).

**Figure 2:**
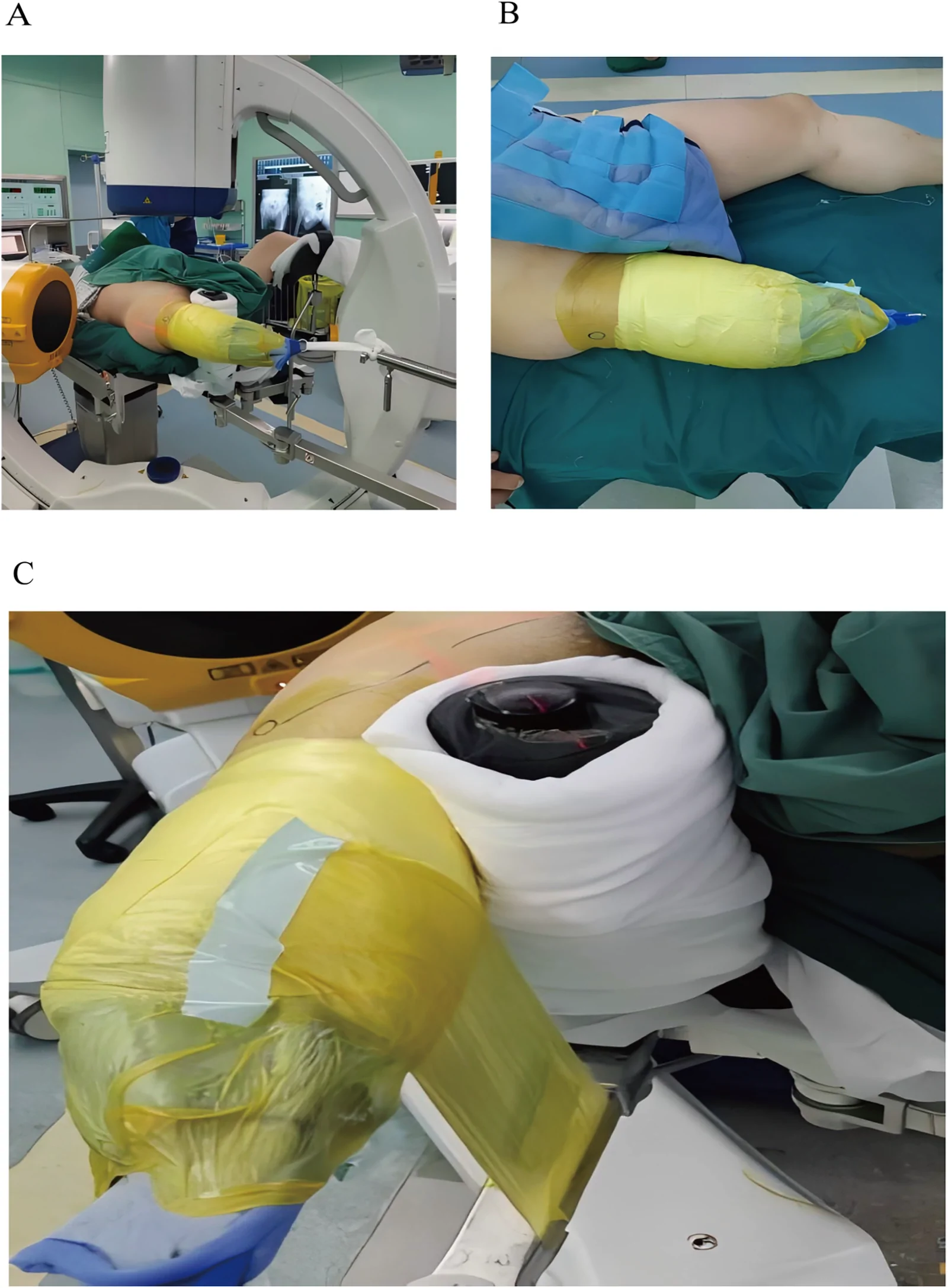
Intraoperative customized traction setup for fracture reduction in the transfemoral amputee patient. **(A)** Overall intraoperative layout: patient positioning on fracture table combined with G-arm fluoroscopy system; **(B)** Lateral view of sterile traction dressing wrapped around the residual stump; **(C)** Close-up detailed view of the traction device for stump rotation control and sustained traction maintenance.

**Figure 3:**
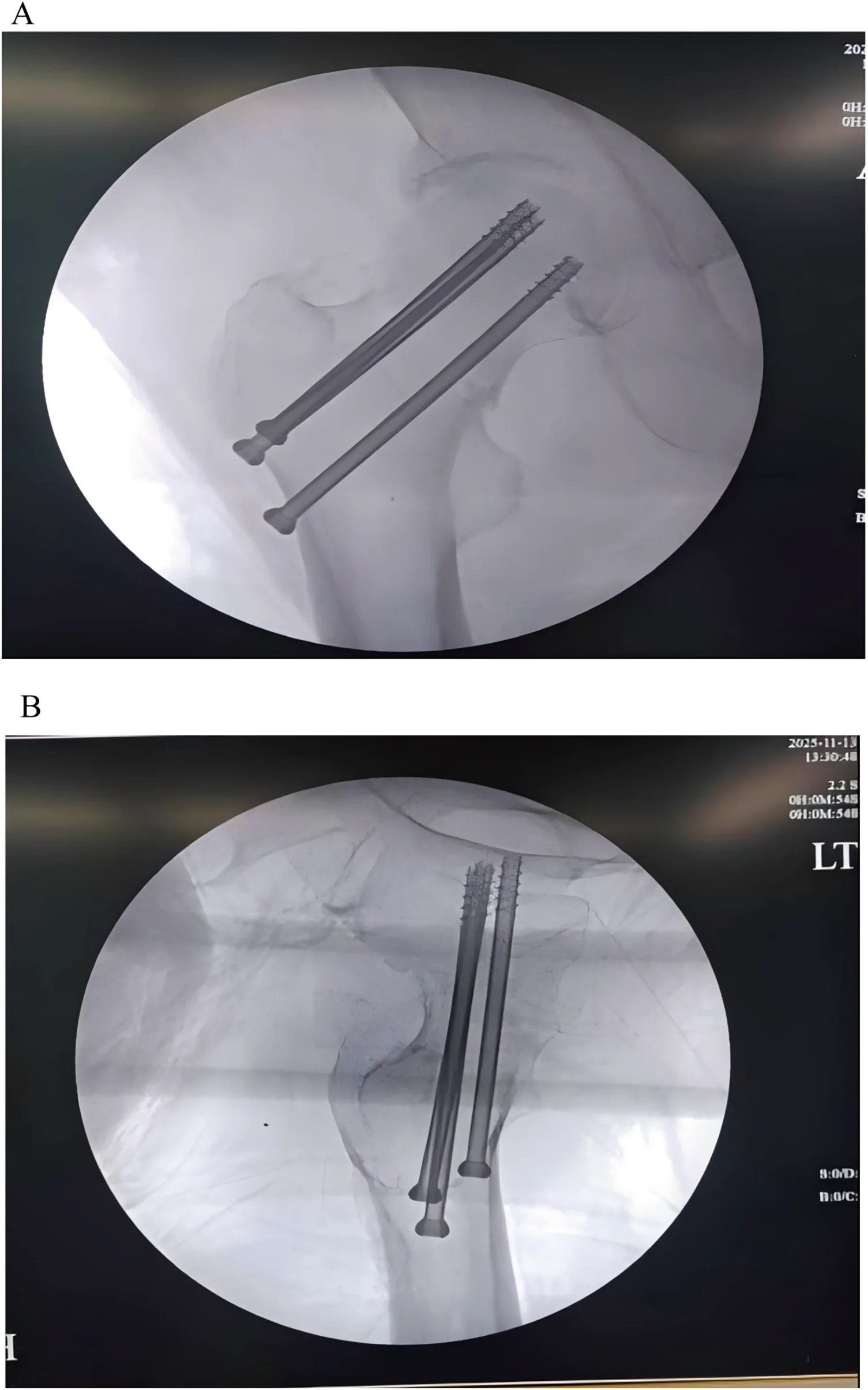
Intraoperative fluoroscopic images after cannulated screw fixation. **(A)** Anteroposterior fluoroscopic view showing three cannulated compression screws arranged in an inverted-triangular configuration; **(B)** Lateral fluoroscopic view confirming satisfactory fracture reduction and implant position. The “LT” marker on this image is an abbreviation for Lateral (G-arm lateral projection), not “Left”. This image corresponds to the patient's right hip.

Intraoperative parameters: operative duration 50 min, intraoperative estimated blood loss 50 mL, fluoroscopy exposure via standard intraoperative fluoroscopic monitoring. Reduction quality was confirmed as anatomical reduction with satisfactory Garden alignment.

Postoperative protocols: weight-bearing restrictions: partial weight-bearing for 6 weeks, with full weight-bearing permitted after radiographic evidence of union; thromboprophylaxis regimen: low-molecular-weight heparin for 14 days. Staged early rehabilitation was implemented postoperatively (detailed rehabilitation protocol: progressive hip range-of-motion exercises followed by partial weight-bearing training).

Follow-up outcomes: total follow-up duration 12 months; fracture union time 6 months. Pre-injury Harris Hip Score: 86 points; Harris Hip Score at final follow-up: 91 points. Final-follow-up pelvic radiographs were obtained (Supplementary Figure S1). No major perioperative or postoperative complications, including stump pressure injury, implant loosening, femoral head osteonecrosis, or non-union, were observed throughout surveillance. Prosthetic-ambulation status at last follow-up: the patient could walk independently using his prosthesis.

### Case 2: primary total hip arthroplasty for Garden IV femoral neck fracture

A 70-year-old male developed acute left-hip pain after a fall while walking with his prosthesis. He sustained left transtibial amputation in a mining accident in 1984 and had relied on prosthetic ambulation for decades. Comorbidities: hypoxemia, osteoporosis; ASA grade: II; bone quality: osteoporotic bone. He retained independent activities of daily living and satisfactory hip function before injury. Time interval from injury to surgery: 7 days. Preoperative pelvic and stump radiographs demonstrated a completely-displaced Garden IV femoral neck fracture (Figure 4). Given the patient's advanced age, severe fracture displacement, and high risks of femoral head avascular necrosis and non-union after internal fixation (6), primary total hip arthroplasty (THA) was selected as definitive treatment.

**Figure 4:**
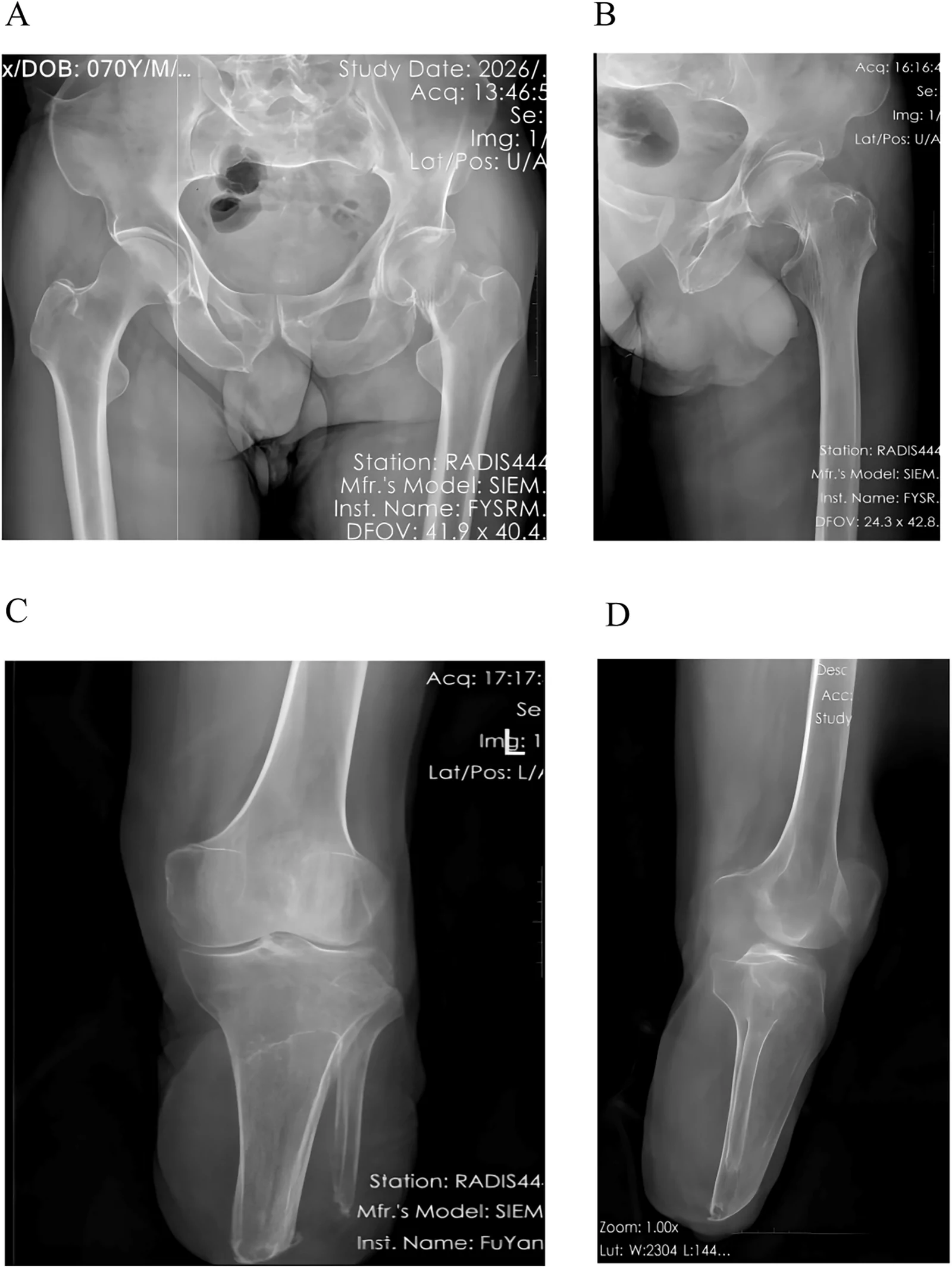
Preoperative radiographic images of case 2 (elderly patient with transtibial amputation complicated with Garden IV femoral neck fracture). **(A,B)** Anteroposterior and lateral pelvic radiographs showing complete displacement of the left femoral neck fracture; **(C,D)** Radiographs of bilateral lower limbs demonstrating pre-existing left transtibial amputation.

Surgery was performed through the posterolateral hip approach with meticulous preservation of periarticular soft tissues.

Intraoperative data: operative time 90 min, estimated blood loss 100 mL. Implant details: prosthesis fixation mode: uncemented, bearing surface: ceramic-on-polyethylene, femoral head size: 28 mm, component positioning parameters: standard safe acetabular cup orientation, offset restoration: anatomical offset reconstruction, soft-tissue repair procedures: complete capsular repair, dislocation prevention measures: posterior capsular reconstruction.

Intra-operative residual-limb management: sterile coverage and stable fixation of the residual stump; intra-operative leg-length discrepancy assessment was performed using bilateral-limb comparison.

Postoperative radiographs confirmed accurate positioning of the hip prosthesis (Figure 5D). Post-operative thromboprophylaxis with low-molecular-weight heparin for 14 days and detailed early-mobilization rehabilitation protocols were implemented. Early weight-bearing and mobilization were encouraged. Total follow-up duration: 6 months. Pre-injury Harris Hip Score: 89 points; Harris Hip Score at final follow-up: 92 points. Final-follow-up radiographs were acquired. The patient achieved favorable prosthetic readaptation and functional recovery during rehabilitation (10). Prosthetic-ambulation status at last follow-up: the patient could ambulate independently with his prosthesis. No major perioperative or postoperative complications were documented.

**Figure 5:**
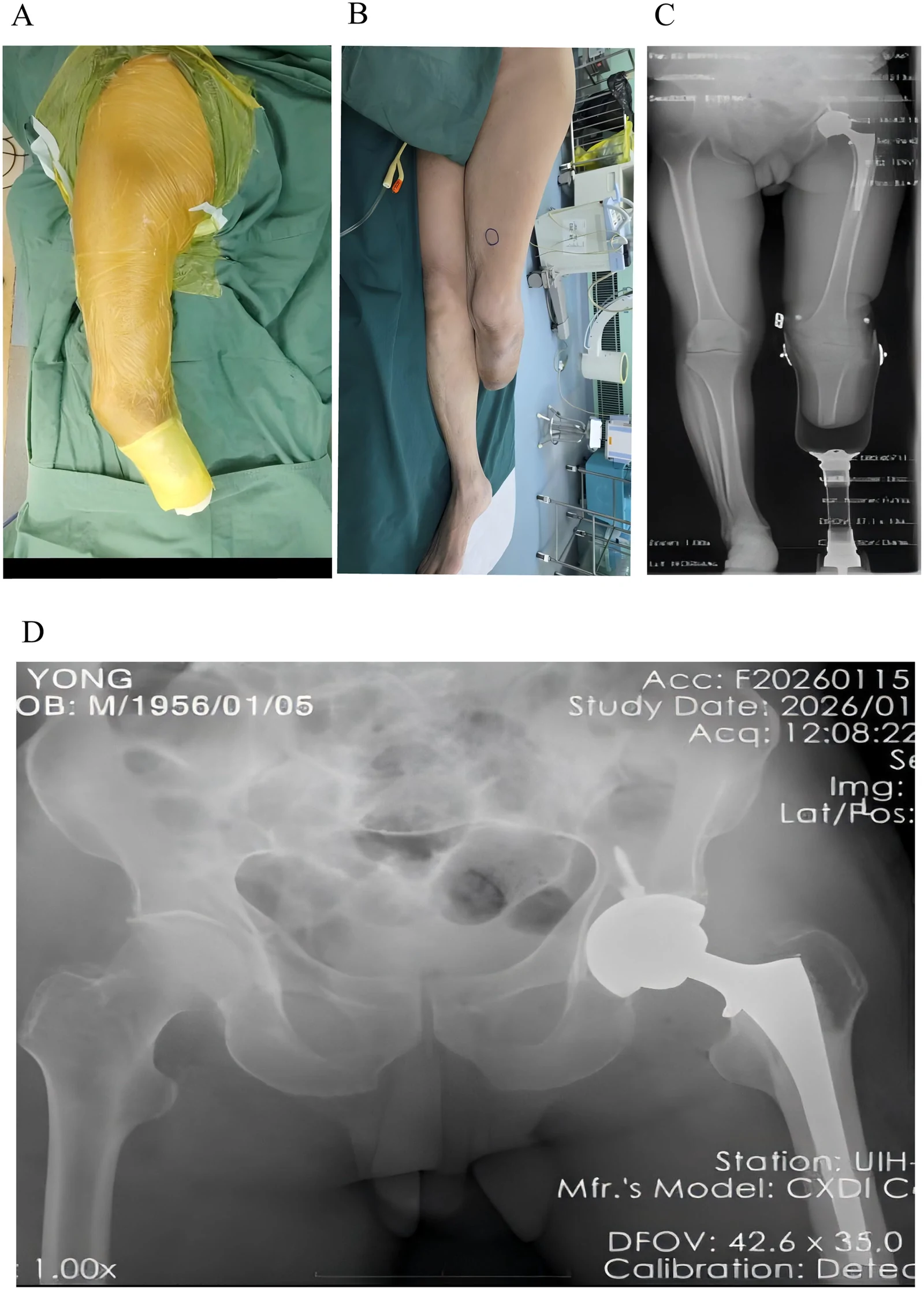
Intraoperative and postoperative images of total hip arthroplasty in case 2. **(A)** Intraoperative sterile draping and disinfection of the affected hip and residual transtibial stump; **(B)** Preoperative skin marking for posterolateral hip surgical approach over the patient's lateral hip; **(C)** Full-length bilateral-lower-limb radiograph showing the patient's permanent transtibial prosthesis before trauma; **(D)** Postoperative anteroposterior pelvic radiograph verifying accurate placement of the total hip prosthesis.

## Discussion

Femoral neck fractures in patients with prior lower-limb amputation represent an uncommon yet surgically-demanding orthopedic condition. Key pathological features include aberrant hip load transmission and reduced bone mass. Early mobilization is essential to mitigate risks of deep-vein thrombosis, pulmonary infection, and permanent prosthetic intolerance (3, 4).

Our first case demonstrates that CCS internal fixation remains a viable treatment for appropriately-selected amputee patients, particularly younger individuals with minimally-displaced fractures and preserved pre-injury hip function. When anatomical reduction is achieved intraoperatively, screw fixation preserves native hip anatomy and minimizes disruption to femoral-head blood supply (8, 9).

Conversely, THA offers distinct advantages for elderly amputees with completely-displaced femoral neck fractures. Internal fixation carries substantial risks of delayed union, non-union, and avascular necrosis in this patient population, which may necessitate revision surgery and delay prosthetic-based rehabilitation. THA provides immediate mechanical stability and allows early weight-bearing — a critical benefit for patients dependent on prosthetic ambulation (10).

Combining our two clinical observations with published evidence, we propose a stratified treatment algorithm derived exclusively from our single-center experience: CCS fixation may be preferred for physiologically younger patients presenting with Garden I-II minimally-displaced fractures in whom satisfactory intraoperative reduction can be achieved (8, 9). THA may be considered for elderly patients with Garden III–IV fully-displaced fractures, where early functional recovery is prioritized and the risk of fixation failure is elevated (2, 6, 7). This decision-making model emphasizes personalized management rather than universal protocols defined solely by amputation status.

## Limitations and future perspectives

This study is limited by its small sample size, which reflects the extremely low incidence of this combined clinical presentation (5). Direct comparative analysis of surgical outcomes between the two interventions cannot be performed owing to inter-case heterogeneity. No definitive conclusions regarding the superiority of either surgical strategy can be drawn from this limited case series. Current literature remains scarce with respect to long-term data on implant survival, fracture union, and sustained prosthetic use. Multicenter registry-based studies with stratified subgroup analyses (age, amputation level, fracture classification) and prolonged functional follow-up are required to develop evidence-based treatment guidelines for this special patient population.

## Conclusions

Femoral neck fractures in patients with prior lower-limb amputation require individualized surgical planning. Based on our limited case-series experience, cannulated compression screw fixation may be appropriate for younger patients with minimally-displaced fractures, whereas primary THA may confer favorable functional outcomes for elderly patients with completely-displaced fractures. Comprehensive preoperative assessment and tailored intraoperative techniques are essential for optimizing clinical outcomes in this rare, high-challenge patient cohort.

## Data Availability

The raw data supporting the conclusions of this article will be made available by the authors, without undue reservation.

## Supplementary material

Supplementary Figure S1 Final-follow-up radiographs for Case 1 and Case 2. Final-follow-up pelvic radiographs of the two patients. (A) Case 1 at 12-month follow-up shows complete fracture union and stable position of cannulated compression screws. (B) Case 2 at 6-month follow-up demonstrates satisfactory positioning of the total hip arthroplasty prosthesis.
